# Expression of P450arom and Estrogen Receptor Alpha in the Oviduct of Chinese Brown Frog (*Rana dybowskii*) during Prehibernation

**DOI:** 10.1155/2015/283085

**Published:** 2015-02-23

**Authors:** Ji Weng, Yuning Liu, Ying Xu, Ruiqi Hu, Haolin Zhang, Xia Sheng, Gen Watanabe, Kazuyoshi Taya, Qiang Weng, Meiyu Xu

**Affiliations:** ^1^College of Biological Science and Technology, Beijing Forestry University, Beijing 100083, China; ^2^Laboratory of Veterinary Physiology, Tokyo University of Agriculture and Technology, Tokyo 183-8509, Japan

## Abstract

One specific physiological phenomenon of Chinese brown frog (*Rana dybowskii*) is that its oviduct expands prior to hibernation instead of expanding during the breeding period. In this study, we investigated the expression of P450arom and estrogen receptors *α* and *β* (ER*α* and ER*β*) in the oviduct of *Rana dybowskii* during the breeding period and prehibernation. The results of the present study showed that there were significant differences in both oviductal weight and size with values markedly higher in prehibernation than in the breeding period. P450arom was observed in stromal tissue in both the breeding period and prehibernation. ER*α* was expressed in stromal tissue and epithelial cells in both periods, whereas ER*β* could not be detected. The mean protein and mRNA levels of P450arom and ER*α* were significantly higher in prehibernation as compared to the breeding period. Besides, oviductal content of 17*β*-estradiol was also higher in prehibernation than in the breeding period. These results suggested that estrogen may play autocrine/paracrine roles mediated by ER*α* in regulating the oviductal hypertrophy during prehibernation.

## 1. Introduction

The female reproductive tract exhibits alterations in structure and function developmentally as well as with seasonal reproductive activity. Oviducts respond to hormonal cues from ovaries with tissue proliferation and differentiation in preparation of transporting and fostering gametes [1]. These responses produce oviductal microenvironments conducive to reproductive success [2]. Oviduct, used in the comparative sense meaning structures derived from the embryonic Müllerian duct, responds to endocrine signals through changes in gene expression, protein synthesis, and morphology with sexual maturation and reproductive activity [3, 4]. Sex steroid hormone receptors in the oviduct receive endocrine signals and regulate growth, differentiation, and protein secretion. Ligand binding of oviductal sex steroid hormone receptors results in a positive feedback that elevates expression levels of sex steroid hormone receptors, priming the tissue to receive further signals in general [5].

Aromatase is a member of the cytochrome P450 superfamily that catalyzes the conversion of androgens (C19), namely, testosterone and androstenedione, into estrogens (C18), estradiol, and estrone, respectively. The enzyme is active in various tissues in both females and males, which means that estrogens are produced not only in gonads but also in extragonadal localizations such as brain, adipose tissue, breast, skin, and bone [6]. Estrogen actions are mediated by two distinct estrogen receptors, estrogen receptor alpha (ER*α*) and estrogen receptor beta (ER*β*), both of which regulate the expression of a variety of different genes [7, 8]. In many tissues such as ovary, placenta, brain, and testis, the key genes of estrogen signaling and biosynthesis,* ESR1* (ER*α*) and* ESR2* (ER*β*), and* Cyp19* that encodes the enzyme P450arom are coexpressed, suggesting that estrogen acts locally as an autocrine or paracrine factor [9–11]. Disruption of these genes demonstrated that estrogen signaling is not only important for the development and differentiation in the reproductive system, but also in nonreproductive systems [12–16].

The Chinese brown frog (*Rana dybowskii*) is an endemic amphibian of northeastern China and has been used widely in the traditional Chinese medicine (TCM) [17]. The hibernation for* Rana dybowskii* takes place from October to February, which is followed by the breeding period ranging from February to June depending on the latitude and altitude. Interestingly, one specific physiological phenomenon that occurs in* Rana dybowskii* is that its oviduct goes through expansion prior to hibernation but not during the breeding period. Our previous studies have shown that PPAR*γ*2, leptin, leptin receptor, the* c-kit*, and proliferating cell nuclear antigen (PCNA) have higher expressions in the oviduct of* Rana dybowskii* during prehibernation compared with the breeding period [18, 19]. To extend our understanding of the regulation of oviductal hypertrophy in* Rana dybowskii*, the present study investigated the expression of P450arom, ER*α*, and ER*β* in the oviduct of* Rana dybowskii* during the breeding period and prehibernation to elucidate the relationship between estrogen and oviductal hypertrophy during prehibernation.

## 2. Materials and Methods

### 2.1. Animals

40 adult female Chinese brown frogs were obtained in April (the breeding period, *n* = 20) and October (prehibernation, *n* = 20) 2013 from Jilin Baekdu Mountain Chinese Brown Frog Breeding Farm, Jilin province (125°40′E-127°56′E, 42°31′N-44°40′N), China. All these animals were treated in accordance with the National Animal Welfare Legislation. All experimental procedures were carried out in accordance with the guidelines established by the Beijing Forestry University. Both the left and right oviducts were collected from* Rana dybowskii*, and the weight of oviduct was measured after necropsy. One side of oviduct was immediately fixed for 24 hours in 4% paraformaldehyde (Sigma Chemical Co., St. Louis, MO, USA) in 0.05 M PBS, pH 7.4 for histological and immunohistochemical observations; the other side of oviduct was immediately stored at −80°C for western blotting, hormonal analysis, and Real-Time PCR detection.

### 2.2. Histology

The oviduct samples were dehydrated in ethanol series and embedded in paraffin wax. Serial sections (4–6 *μ*m) were mounted on slides coated with poly-L-lysine (Sigma). Some sections were stained with hematoxylin and eosin (HE) for observations of general histology.

### 2.3. Immunohistochemistry

The serial sections of 6 randomly chosen oviduct tissues of each period were heated in a heat-induced epitope retrieval buffer and were naturally cooled down. 3% hydrogen peroxide buffer was used for endogenous peroxidase blocking before the sections were incubated with 10% normal goat serum to reduce background staining caused by the second antibody. For our studies we used rabbit anti-human polyclonal antibodies. The sections were then incubated with rabbit anti-human P450arom (H-300) (Santa Cruz Biotechnology, Santa Cruz, CA, USA), rabbit anti-human ER*α* (MC-20) (Santa Cruz), and rabbit anti-human ER*β* (H-150) (Santa Cruz) overnight at 4°C. The specificity of the polyclonal antibodies against human P450arom, ER*α*, and ER*β* was evaluated by preadsorption (overnight at 4°C) of the primary antibodies with the human recombinant proteins P450arom, ER*α*, and ER*β* at a molar ratio of 1 : 10. The sections were then incubated with a second antibody, goat anti-rabbit IgG conjugated with biotin and peroxidase with avidin, using a rabbit ExtrAvidin staining kit (Sigma) followed by visualizing with 30 mg 3,3-diaminobenzidine (Wako, Tokyo, Japan) solution in 100 mL of 0.05 mol Tris-HCl buffer, pH 7.6, plus 30 *μ*L H_2_O_2_. Finally, the reacting sections for P450arom, ER*α*, and ER*β* were counterstained with hematoxylin solution (Merck, Tokyo, Japan). To value the specificity of the polyclonal antibodies, P450arom antibodies were performed in the testes of rats, and ER*α* and ER*β* antibodies were in the nuptial pad of male* Rana dybowskii*, which are known to express these proteins. The specificity of the ER*α*, ER*β*, and P450arom also has been described for muskrats [14] and wild ground squirrel [20] reported by our research team previously.

### 2.4. Western Blotting

Oviductal tissues (9 samples of each period) were weighed and diced into small pieces using a clean razor blade, respectively. The tissue was homogenized in a homogenizer containing 300 *μ*L of 10 mg/mL PMSF stock and incubated on ice for 30 min throughout all the procedures. Homogenates were centrifuged at 12,000 g for 10 min at 4°C. Protein extracts (25 *μ*g) were mixed with an equal volume of 2x Laemmli sample buffer. Equal amounts of each sample were loaded and run on a 12% SDS-PAGE gel at 18 V/cm and transferred to nitrocellulose membranes using a wet transblotting apparatus (Bio-Rad, Richmond, CA, USA). The membranes were blocked in 3% BSA for 1 hour at room temperature. Primary incubation of the membranes was carried out using a 1 : 1000 dilution of rabbit anti-human placental P450arom antibody, rabbit anti-human ER*α*, and rabbit anti-human ER*β* for 60 min. Secondary incubation of the membrane was then carried out using a 1 : 1000 dilution of goat anti-rabbit IgG tagged with horseradish peroxidase for 60 min. Finally, the membrane was colored with 10 mg 3,3-diaminobenzidine (Wako) solution in 50 mL phosphate buffer (0.03 M) plus 3 *μ*L H_2_O_2_. For the negative control, the preabsorbed primary antibodies with respective antigens were used instead of primary antibodies and another procedure is the same as described above. The intensities of the bands were quantified using Quantity One software (Version 4.5, Bio-Rad Laboratories).

### 2.5. Real-Time PCR Analysis

Total RNA was extracted from 9 oviductal tissues of each period using TRIzol Reagent (Invitrogen Co., CA, USA) according to the protocol. The mRNA expressions of* ERα*, ER*β* , and* Cyp19* were analyzed by Real-Time PCR using one-step SYBR PrimeScript RT-PCR kit (Table 1). Tissues dissected from 3 to 10 individuals were pooled from* Rana dybowskii* to analyze expression in oviducts. The primers for Real-Time PCR analysis were designed using the Primer 3 program [21, Table 1]. The PCR reactions were carried out in a 20 *μ*L volume and performed with ABI PRISM 7500 Fast Real-Time PCR System (Applied Biosystems, Foster City, CA) using the following conditions: reverse transcription at 42°C for 5 min and 95°C for 30 sec, followed by PCR reaction of 40 cycles at 95°C for 5 sec and 60°C for 34 sec and dissociation protocol. Transcript levels of the target genes were normalized to the *β*-actin after correcting for differences in amplification efficiency. The expression level of each target mRNA relative to *β*-actin mRNA was determined using the 2^−ΔΔCt^ method.

### 2.6. Sequence Analysis

DNA sequence was determined using the ABI-PRISM 3730 sequencer (Invitrogen). Searching of similar sequences was performed using BLASTP in the nonredundant (nr) protein sequences database of the NCBI website.

### 2.7. Hormone Assay

The oviductal content of 17*β*-estradiol was determined by radioimmunoassay (RIA) kits (Kit 02010306021 for 17*β*-estradiol, China Diagnostics Medical Corporation, Beijing, China) that are designed for the detection of rat hormone. The intra-assay variation was less than 10% for 17*β*-estradiol. The detection sensitivity was 5 pg/mL for 17*β*-estradiol.

### 2.8. Statistical Analysis

Statistical comparisons were made with Student's* t*-test. A value of *P* < 0.05 was considered as an indication of statistical significance.

## 3. Results

### 3.1. Changes in Oviductal Histology between the Breeding Period and Prehibernation

**Figure 1:**
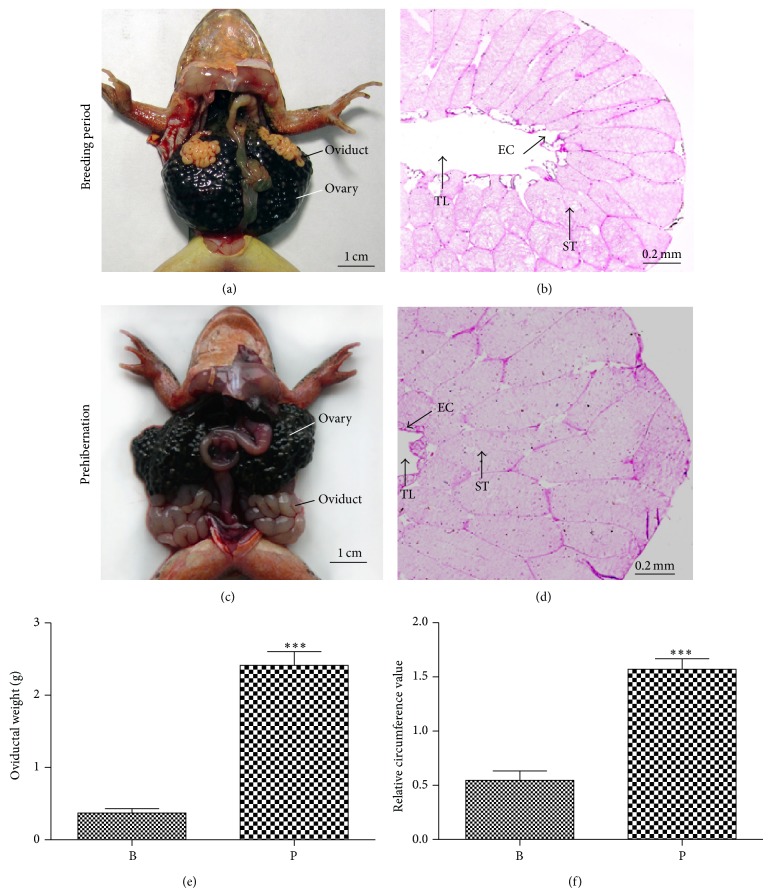
Changes in oviductal histology between the breeding period and prehibernation. Anatomic localization and morphology of oviduct of* Rana dybowskii* during the breeding period (a) and prehibernation (c). Histological structure of* Rana dybowskii* oviducts by hematoxylin and eosin staining in the breeding period (b) and prehibernation (d). Tubule lumen, epithelial cells, and lobules consist of stroma cells and were observed in oviduct tissues expanded. Changes in weight (e) and relative circumference (f) of oviduct in* Rana dybowskii* from the breeding period to prehibernation. B: the breeding period; P: prehibernation; EC: epithelial cell; ST: stroma tissue; TL: tubule lumen. Scale bars represent 1 cm ((a), (c)) and 0.2 mm ((b), (d)), respectively.

Anatomic and morphologic observations of oviduct were present in the female* Rana dybowskii* during the breeding period and prehibernation (Figures 1(a) and 1(c)). The histological appearances of oviductal tissues from the breeding period and prehibernation were shown in Figures 1(b) and 1(d). The oviduct included tubule lumen, epithelium, and lobules that consist of stromal cells. The higher oviductal weight and relative circumference values of the oviduct were observed during prehibernation in October and lower values were found during breeding period in April (Figures 1(e) and 1(f)).

### 3.2. Immunolocalization of P450arom, ER*α*, and ER*β* in* Rana dybowskii* Oviduct

**Figure 2:**
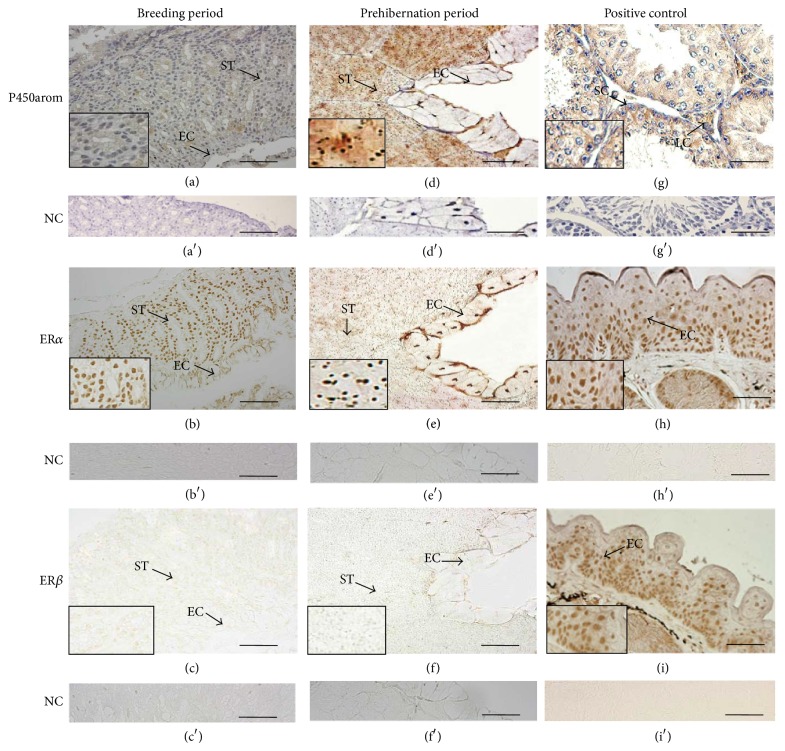
Immunolocalization of P450arom, ER*α*, and ER*β* in the oviduct of* Rana dybowskii* during the breeding period and prehibernation. Positive immunostaining of P450arom was observed in the cytoplasm in both epithelial and stromal cells of the breeding period and prehibernation ((a), (d)), and stronger positive staining was shown in prehibernation (d). Immunoreaction for ER*α* was present in the nucleoli of both epithelial and stromal cells of the breeding period and prehibernation ((b), (e)), while the immunoreactivity for ER*β* was not found ((c), (f)). P450arom was detected in Sertoli cells and Leydig cells in the testes of rat as positive control (g), while ER*α* and ER*β* were detected in the nucleus of epithelial cell of the nuptial pad of male* Rana dybowskii* ((h), (i)). No immunostaining was detected in negative control sections in which the primary antibodies were preabsorbed by respective antigens ((a′)–(i′)). EC: epithelial cells; SC: Sertoli cells; LC: Leydig cells; NC: negative control. Scale bars represent 50 *μ*m.

Immunohistochemical staining of P450arom, ER*α*, and ER*β* was performed in oviduct of* Rana dybowskii* during the breeding period and prehibernation (Figure 2). Positive signal of P450arom was localized in the cytoplasm in both epithelial and stromal cells of the breeding period and prehibernation (Figures 2(a) and 2(d)). Immunohistochemical reaction for ER*α* was observed in the nucleoli of both epithelial and stromal cells of the breeding period and prehibernation (Figures 2(b) and 2(e)). Stronger immunostainings for P450arom and ER*α* were present in oviduct during prehibernation. Unexpectedly, the immunoreactivity for ER*β* was not found in oviduct of* Rana dybowskii* either during the breeding period or during prehibernation (Figures 2(c) and 2(f)). For positive control, P450arom was detected in Sertoli cells and Leydig cells in the testes of rat (Figure 2(g)), while ER*α* and ER*β* were detected in the nucleus of epithelial cell of the nuptial pad of male* Rana dybowskii*  (Figures 2(h) and 2(i)). Sections treated with preabsorption of primary antibody were used as negative controls and were shown in Figures 2(a′)–2(i′). A substantial decrease in tissue immunostaining was observed after preabsorption of the rabbit anti-human P450arom, ER*α*, and ER*β* antibodies with the specific recombinant proteins (Figures 2(a′)–2(i′)).

### 3.3. Expression of P450arom, ER*α*, and ER*β* Proteins

**Figure 3:**
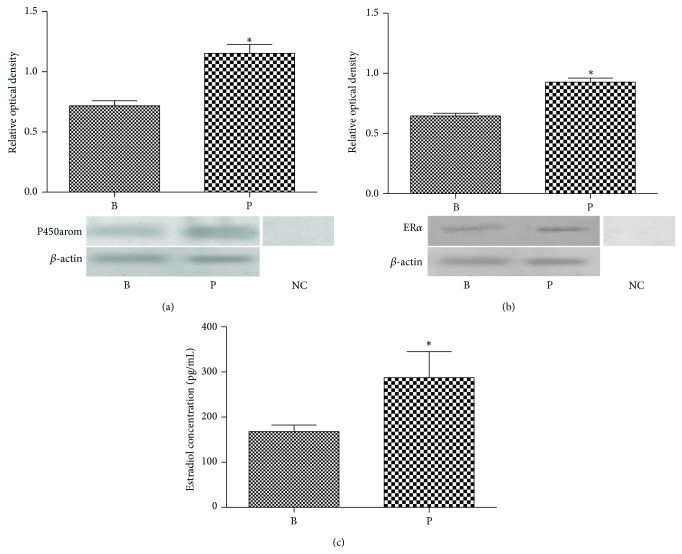
Western blotting of P50arom and ER*α* in the oviduct tissues of* Rana dybowskii* during the breeding period (B) and prehibernation (P). The oviductal content of estradiol-17 during the breeding period (B) and prehibernation (P). The positive bands of P450arom and ER*α* were observed in the position of about 55 kDa (a) and 66 kDa (b), respectively. *β*-actin blots were used as controls to correct for loading in each lane. The preabsorbed primary antibody was used instead of primary antibody for the negative control (lane NC). The estradiol-17 concentration was detected in both periods (c). NC: negative control. The expression levels were determined by densitometric analysis. Bars represent mean ± SD for three independent experiments. Mean values within the columns marked with asterisk were used instead of letters to indicate significant difference (*P* < 0.05).

Western analysis of proteins extracted from the* Rana dybowskii* oviductal tissue samples revealed immunoreactive P450arom and ER*α* proteins at 55 kD and 66 kD, respectively, during the breeding and prehibernation periods (Figure 3). The results were normalized to the expression level of *β*-actin. The expressions of P450arom and ER*α* were significantly high in prehibernation as compared to the breeding period (Figures 3(a) and 3(b)). The immunoreactivity for ER*β* was not found in oviductal tissues of* Rana dybowskii* in the breeding period and prehibernation (data not shown). The primary antibodies preabsorbed with an excess amount of the antigens were used for the negative control (Figures 3(a) and 3(b), lane NC).

### 3.4. The Oviductal Content of 17*β*-Estradiol

The level of the oviductal content of 17*β*-estradiol was shown in Figure 3(c) (*P* < 0.01). The oviductal 17*β*-estradiol concentration was significantly high in prehibernation (287.6 ± 25.68 pg/mL) as compared to the breeding period (167.8 ± 7.371 pg/mL).

### 3.5. Expressions of P450arom and ER*α* mRNA

**Figure 4:**
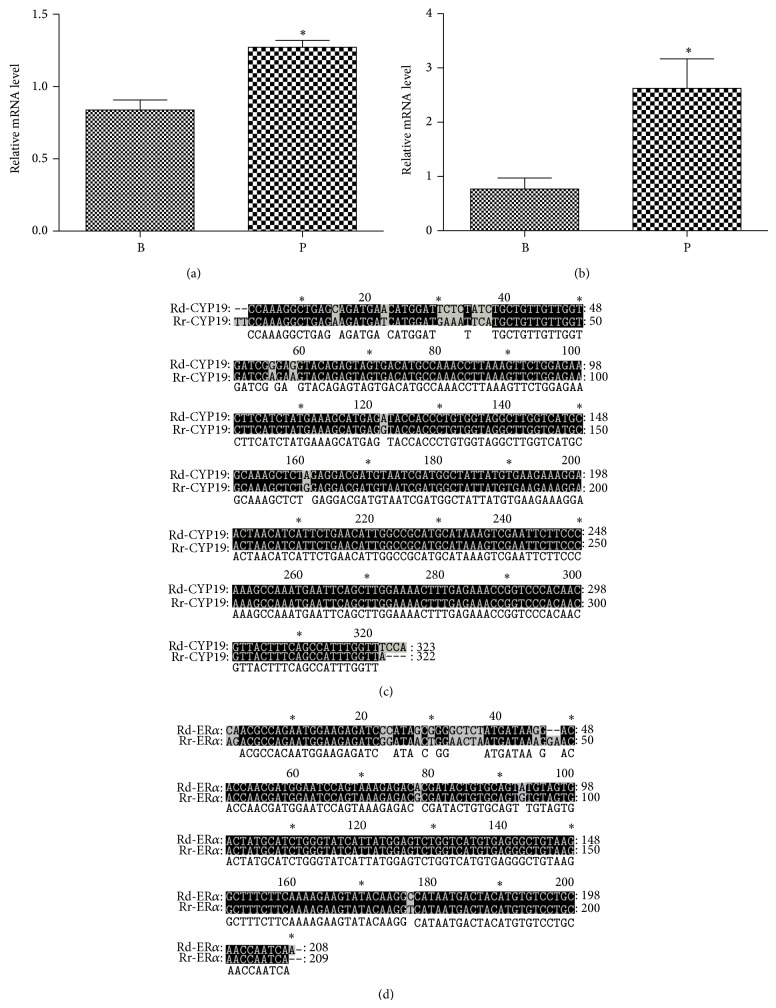
The relative* CYP19* (a) and* ERα* (b) mRNA in the oviduct of* Rana dybowskii* during the breeding period (B) and prehibernation (P). Bars represent mean ± SD for three independent experiments. Mean values within the columns marked the same as above indicate significant difference (*P* < 0.05). Compared with* Rana rugosa (Rr)*, the cDNA nucleotide sequence identity was 91.43% for* Cyp19* (c) and 94.15% for* ERα* (d).

*Cyp19* (Figure 4(a)) and ER*α* (Figure 4(b)) mRNA levels were detected in oviductal tissues of* Rana dybowskii* during the breeding period and prehibernation. The primers specific for* Cyp19* was used to amplify a Real-Time PCR product of 304 bp, while production of 180 bp for ER*α*. Meanwhile, the mRNA expressions of these two genes were generally in line with those of their corresponding proteins. Real-time PCR analysis revealed a significant increase in* Cyp19* and* ERα* mRNA levels during prehibernation as compared to the breeding period.* ERβ* gene was not expressed in oviductal tissues of* Rana dybowskii* in the breeding period and prehibernation (data not shown). To further confirm the nature of the signals, cDNA fragments for* Cyp19* and* ERα* in oviductal tissues of* Rana dybowskii* were sequenced and compared to the corresponding fragments in* Rana rugosa*. The 304 bp* Cyp19* cDNA nucleotide sequence identity is 94.15% (Figure 4(c)) and the 180 bp* ERα* cDNA nucleotide sequence identity is 91.43% (Figure 4(d)).

## 4. Discussion

This is the first study to investigate the expression of P450arom, ER*α*, and ER*β* in oviduct of* Rana dybowskii*, which clearly showed that the expression patterns of P450arom and ER*α* were correlated with the changes of* Rana dybowskii* oviductal content of 17*β*-estradiol during the breeding period and prehibernation. These findings emphasize local estrogen synthesis in oviduct of* Rana dybowskii* and also emphasize that estrogen may play an important autocrine or paracrine regulatory roles in oviductal hypertrophy during prehibernation.

Several experimental models have shown that oviductal development is regulated by the sex steroid hormones, estrogen and progesterone [22]. Estrogens control development of sex accessory structures such as the hypertrophy of oviduct prior to sexual maturation and during each season prior to ovulation [23]. In the* Rana dybowskii*, the oviductal hypertrophy took place during prehibernation instead of the breeding period. Epithelial cells and a large number of stromal cells were observed in oviductal tissues of prehibernation. Moreover, significantly high values of oviductal weight and size (relative circumference value) were also found in prehibernation when compared to those of the breeding period. These findings implied that the hypertrophy of oviduct might be speculated where locally produced intrinsic regulators might play a regulatory role in the oviductal hypertrophy of* Rana dybowskii*.

Estrogens synthesized locally in extragonadal sites mostly do not enter the circulation but exert intracrine, autocrine, or paracrine and juxtacrine effects, acting directly in the cells of synthesis or on the neighbouring cells [24]. In the present study, P450arom was expressed in oviductal tissues of* Rana dybowskii*, showing that* Rana dybowskii* oviduct may be able to secrete estrogen locally. These findings were not unique to* Rana dybowskii*, as similar results were found in the oviduct of the northern leopard frog,* Rana pipiens*, which showed that androgen was aromatized to estrogen by the oviduct and suggested that this conversion might account for its growth-promoting effect [25]. Similar phenomenon was also observed in the epithelial cells of the fimbriae during tubal pregnancy of human compared to normal fallopian tube specimens, which may result in a sufficient local estrogen supply for the maintenance of tubal pregnancy [26]. In addition, in bovine oviduct primary cell culture system, it was reported that the cell line maintains some important characteristics of the primary cells such as the expression of estrogen receptors and P450arom, showing that androgen can be aromatized in the oviduct to local estrogens [27]. These findings suggested a possible local production of estrogen that, acting in an autocrine or paracrine fashion, may promote oviductal function.

In mammals, estrogens play a role in folliculogenesis and in the maintenance of the ovarian somatic cells. However, in some fish, amphibians, and reptiles, estrogens are associated not only with ovarian development as in mammals, but also with sex determination and reproductive organs differentiation. In* Xenopus*, higher aromatase mRNA level in the* Xenopus* brain than in other tissues occurs irrespective of sex differences, and the protein level is detected in the early stages of brain morphogenesis, which showed that, besides its role in neural development, 17*β*-estradiol in the brain acts to protect against damage from neuronal injury and disease [28]. In the chick and mouse oviduct, 17*β*-estradiol induced epithelial cell proliferation and differentiation [29]. The differentiation of the secretory epithelial cell phenotype into the ciliated epithelial cell phenotype can be induced by 17*β*-estradiol in primary cultured human fallopian tube epithelial cells [30]. Study in oviducts of* Rana cyanophlyctis* also indicated that 17*β*-estradiol injections induced oviductal hypertrophy, and in androgen treated frogs there was also an increase in the oviductal dry weight and protein content [31]. Therefore, the present study provides evidence that expression patterns of P450arom and ER*α* were correlated with the changes of oviductal content of 17*β*-estradiol during the breeding period and prehibernation, suggesting that estrogens, in an autocrine or paracrine manner, might mediate the oviductal hypertrophy of* Rana dybowskii* during prehibernation.

Clearly, estrogens exert strong effects on female reproductive events, which are principally mediated by the classical ERs, ER*α* and ER*β* [7]. Both ER*α* and ER*β* regulate the expression of a variety of different genes. In the present study, only ER*α* was present in oviductal tissues of* Rana dybowskii*, suggesting that estrogen signaling through ER*α* subtypes involved in modulating oviductal function in seasonal changes. Similarly, in* Xenopus laevis*, highly conserved domains of the ER*α* and mRNA products of the predicted size were amplified from laryngeal muscle, forebrain, and oviduct [32]. These findings are also consistent with studies of other species. In developing mice and rats, ER*α* is expressed in oviductal epithelial and stromal cells, but ER*β* is absent in both cell types [33, 34]. Pre- and neonatal exposure to diethylstilbestrol (DES), a synthetic estrogen, caused abnormal morphology and altered cell proliferation in mouse and rat oviducts [35, 36]. Taken together, our present results were in agreement with the view that differentiation and proliferation of epithelial cells in the oviduct may be regulated by estradiol through ER*α* [37].

In conclusion, to our knowledge, this was the first comprehensive report on the expression of P450arom and ER*α* in oviductal tissues of* Rana dybowskii* during the breeding period and prehibernation. The present results suggested that oviduct might be able to potentially synthesize estrogen, which could potentially act locally in an autocrine/paracrine manner via ER*α*, in order to exert a regulatory role in the oviductal hypertrophy of* Rana dybowskii* during prehibernation.

## Figures and Tables

**Table 1:** Oligonucleotide primers used for quantitative Real-Time PCR.

Gene name	Primer sequence	Product size (bp)
*ERα *	AGACGCCAGAATGGAAGAGA (forward) TGATTGGTTGCAGGACACAT (reverse)	209

*ERβ *	CCGGGAAGTCATGAAGAGAA (forward) GTCAATGGTGCACTGGTTTG (reverse)	

*Cyp19 *	TCCAAAGGCTGAGAAGATGA (forward) GAAACCAAATGGCTGAAAGT (reverse)	322

*β-actin *	TTGCTGATCCACATCTGCT (forward) GACAGGATGCAGAAGGAGAT (reverse)	146

## References

[1] Moore B. C., Forouhar S., Kohno S., Botteri N. L., Hamlin H. J., Guillette L. J. (2012). Gonadotropin-induced changes in oviducal mRNA expression levels of sex steroid hormone receptors and activin-related signaling factors in the alligator. *General and Comparative Endocrinology*.

[2] Weng Q., Shi Z., Watanabe G., Taya K. (2009). Immunolocalization of NGF and its receptors trkA and p75 in the oviducts of golden hamsters during the estrous cycle. *Experimental Animals*.

[3] Jefferson W. N., Padilla-Banks E., Goulding E. H., Lao S.-P. C., Newbold R. R., Williams C. J. (2009). Neonatal exposure to genistein disrupts ability of female mouse reproductive tract to support preimplantation embryo development and implantation. *Biology of Reproduction*.

[4] Leese H. J. (1988). The formation and function of oviduct fluid. *Journal of Reproduction and Fertility*.

[5] Cunha G. R., Cooke P. S., Kurita T. (2004). Role of stromal-epithelial interactions in hormonal responses. *Archives of Histology and Cytology*.

[6] Czajka-Oraniec I., Simpson E. R. (2010). Aromatase research and its clinical significance. *Endokrynologia Polska*.

[7] Kuiper G. G. J. M., Enmark E., Gustafsson J.-Å., Pelto-Huikko M., Nilsson S. (1996). Cloning of a novel estrogen receptor expressed in rat prostate and ovary. *Proceedings of the National Academy of Sciences of the United States of America*.

[8] Wong M., Thompson T. L., Moss R. L. (1996). Nongenomic actions of estrogen in the brain: physiological significance and cellular mechanisms. *Critical Reviews in Neurobiology*.

[9] Leung S. T., Reynolds T. S., Wathes D. C. (1998). Regulation of oxytocin receptor in the placentome capsule throughout pregnancy in the ewe: the possible role of oestradiol receptor, progesterone receptor and aromatase. *Journal of Endocrinology*.

[10] Tsuruo Y., Ishimura K., Osawa Y. (1995). Presence of estrogen receptors in aromatase-immunoreactive neurons in the mouse brain. *Neuroscience Letters*.

[11] O'Donnell L., Robertson K. M., Jones M. E., Simpson E. R. (2001). Estrogen and spermatogenesis. *Endocrine Reviews*.

[12] Renoir J.-M. (2012). Estradiol receptors in breast cancer cells: associated co-factors as targets for new therapeutic approaches. *Steroids*.

[13] Ghosh S., Choudary A., Musi N., Hu Y., Li R. (2009). IKKbeta mediates cell shape-induced aromatase expression and estrogen biosynthesis in adipose stromal cells. *Molecular Endocrinology*.

[14] Lu L., Zhang H., Lv N. (2011). Immunolocalization of androgen receptor, aromatase cytochrome P450, estrogen receptor alpha and estrogen receptor beta proteins during the breeding season in scent glands of muskrats (*Ondatra zibethicus*). *Zoological Science*.

[15] Peruffo A., Giacomello M., Montelli S., Corain L., Cozzi B. (2011). Expression and localization of aromatase P450AROM, estrogen receptor-*α*, and estrogen receptor-*β* in the developing fetal bovine frontal cortex. *General and Comparative Endocrinology*.

[16] Yu S., Xing X., Jiao K., Sun L., Liu L., Wang M. (2012). Changes in the expression of aromatase, estrogen receptor *α* and *β* in mandibular condylar cartilage of rats induced by disordered occlusion. *BMC Musculoskeletal Disorders*.

[17] Zhang Z., Zhang B., Nie X., Liu Q., Xie F., Shang D. (2009). Transcriptome analysis and identification of genes related to immune function in skin of the Chinese brown frog. *Zoological Science*.

[18] Liu Y., Weng J., Huang S. (2014). Immunoreactivities of PPAR*γ*2, leptin and leptin receptor in oviduct of Chinese brown frog during the breeding period and pre-hibernation. *European Journal of Histochemistry*.

[19] Shen Y., Liu Y., Ma J. (2012). Immunoreactivity of *c-kit* receptor protein during the prehibernation period in the oviduct of the Chinese brown frog, *Rana chensinensis*. *Journal of Veterinary Medical Science*.

[20] Zhang H., Sheng X., Hu X. (2010). Seasonal changes in spermatogenesis and immunolocalization of cytochrome P450 17*α*-Hydroxylase/c17-20 lyase and cytochrome P450 aromatase in the wild male ground squirrel (*Citellus dauricus* Brandt). *The Journal of Reproduction and Development*.

[21] Rozen S., Skaletsky H. (2000). Primer 3 on the WWW for general users and for biologist programmers. *Methods in Molecular Biology*.

[22] Jansen R. P. (1984). Endocrine response in the fallopian tube. *Endocrine Reviews*.

[23] David O. N. (2007). Comparative aspects of vertebrate reproduction. *Vertebrate Endocrinology*.

[24] Harada N., Sasano H., Takagi Y., Murakami H., Ohkuma T., Nagura H. (1999). Localized expression of aromatase in human vascular tissues. *Circulation Research*.

[25] Kobayashi F., Zimniski S. J., Smalley K. N. (1996). Characterization of oviductal aromatase in the northern leopard frog, Rana pipiens. *Comparative Biochemistry and Physiology, B Biochemistry and Molecular Biology*.

[26] Li Y., Qin L., Xiao Z.-J. (2003). Expression of P450 aromatase and 17 beta-hydroxysteroid dehydrogenase type 1 at fetal-maternal interface during tubal pregnancy. *The Journal of Steroid Biochemistry and Molecular Biology*.

[27] Schoen J., Bondzio A., Topp K., Einspanier R. (2008). Establishment and characterization of an adherent pure epithelial cell line derived from the bovine oviduct. *Theriogenology*.

[28] Azcoitia I., Arevalo M.-A., De Nicola A. F., Garcia-Segura L. M. (2011). Neuroprotective actions of estradiol revisited. *Trends in Endocrinology and Metabolism*.

[29] Anderson R. G. W., Hein C. E. (1976). Estrogen dependent ciliogenesis in the chick oviduct. *Cell & Tissue Research*.

[30] Comer M. T., Leese H. J., Southgate J. (1998). Induction of a differentiated ciliated cell phenotype in primary cultures of Fallopian tube epithelium. *Human Reproduction*.

[31] Pancharatna K., Rajapurohit S. V., Hiregoudar S. R., Kumbar S. M. (2001). Effect of androgens on oviductal growth in skipper frog *Rana cyanophlyctis*. *Indian Journal of Experimental Biology*.

[32] Wu K. H., Tobias M. L., Kelley D. B. (2003). Estrogen receptor expression in laryngeal muscle in relation to estrogen-dependent increases in synapse strength. *Neuroendocrinology*.

[33] Yamashita S., Newbold R. R., McLachlan J. A., Korach K. S. (1989). Developmental pattern of estrogen receptor expression in female mouse genital tracts. *Endocrinology*.

[34] Okada A., Ohta Y., Buchanan D. L. (2002). Changes in ontogenetic expression of estrogen receptor alpha and not of estrogen receptor beta in the female rat reproductive tract. *Journal of Molecular Endocrinology*.

[35] Okada A., Sato T., Ohta Y., Buchanan D. L., Iguchi T. (2001). Effect of diethylstilbestrol on cell proliferation and expression of epidermal growth factor in the developing female rat reproductive tract. *The Journal of Endocrinology*.

[36] Newbold R. R., Bullock B. C., Mc Lachlan J. A. (1983). Exposure to diethylstilbestrol during pregnancy permanently alters the ovary and oviduct. *Biology of Reproduction*.

[37] Okada A., Ohta S. L., Brody S. L. (2004). Role of foxj1 and estrogen receptor alpha in ciliated epithelial cell differentiation of the neonatal oviduct. *Journal of Molecular Endocrinology*.

